# Mother–Infant Physiological Coregulation Is Associated With Infant Developmental Status Among Families With Experienced Adversity

**DOI:** 10.1002/dev.70113

**Published:** 2025-12-07

**Authors:** Samantha M. Brown, Erika Lunkenheimer, Savannah A. Girod, Jill T. Krause, C. Nathan Marti, Keri J. Heilman

**Affiliations:** ^1^ Department of Human Development and Family Studies Colorado State University Fort Collins Colorado USA; ^2^ Department of Psychology Pennsylvania State University University Park Pennsylvania USA; ^3^ Department of Human Development and Family Studies Texas Tech University Lubbock Texas USA; ^4^ Abacist Analytics Austin Texas USA; ^5^ Department of Psychiatry University of North Carolina Chapel Hill North Carolina USA

**Keywords:** coregulation, infant development, mother–infant interaction, respiratory sinus arrhythmia, synchrony

## Abstract

Mother–infant physiological coregulation may vary by the infants’ developmental needs. We examined whether infant developmental status was associated with dynamic trajectories of mother–infant respiratory sinus arrhythmia (RSA) coregulation. Fifty‐three US mothers and infants oversampled for lower income, higher stress, and child maltreatment risk completed a double Face‐to‐Face Still‐Face (FFSF) paradigm, alternating between play and stress conditions, in which RSA was collected in 30‐s epochs. Infant development was measured using the Developmental Assessment for Young Children Socioemotional Skills and Adaptive Behavior subscales and the Survey of Well‐Being of Young Children Developmental Milestones subscale. Growth modeling of RSA coregulation across the FFSF indicated two significant interactions: a time × mother RSA × infant socioemotional skills interaction, indicating a dyadic RSA pattern whereby maternal physiological engagement (RSA withdrawal) buffering infant stress (RSA augmentation) was linked to higher infant socioemotional competence; and a condition × infant RSA × developmental milestones interaction, suggesting that in infants with lower developmental milestones, this same dyadic pattern of infant RSA augmentation coupled with maternal RSA withdrawal was more likely during stress conditions. Findings suggest mother–infant RSA coregulation varies by infants’ developmental status, with mothers offering more support during challenging contexts. Findings may inform the promotion of adaptive caregiving behaviors and infant development in interventions with high‐risk families.

## 1 Introduction

Mother–infant biobehavioral coregulation, operationalized in the present study as synchrony, involves moment‐to‐moment reciprocal processes through which parents and children mutually influence and regulate each other's emotions, behaviors, and physiological responses. This concept is gaining significant attention in the field of developmental psychology as a key factor in early development. According to the biobehavioral synchrony model, mothers and infants learn to coordinate their affective, behavioral, and physiological responses through dynamic face‐to‐face interactions (Feldman 2012). Indeed, concordance of mother–infant coregulation (also referred to as positive synchrony) has been identified as a key dyadic process underlying infant socioemotional and regulatory development (Feldman 2007a). However, a limited literature suggests that mother–infant coregulation may function differently for families experiencing adversity. Notably, among high‐risk mothers and children, dyads often exhibit weakened (less positive), absent (no), or discordant (negative) associations between trajectories of mother and infant respiratory sinus arrhythmia (RSA), reflecting disruptions in the coregulation of affect, behavior, and physiology (Brown et al. 2021; Lunkenheimer, Busuito, et al. 2018; Skowron et al. 2013). This lack of synchronization is commonly associated with negative outcomes, such as internalizing and externalizing behaviors and cognitive processing issues (Feldman 2007b; Lunkenheimer, Tiberio, et al. 2018). However, we propose that some dyads exposed to adversity may display distinct patterns of physiological coregulation that reflect resilience or adaptive functioning, particularly in different caregiving contexts.

Researchers have emphasized the importance of predictable caregiving for child development, and caregiving that is inconsistent or unpredictable is often found in adverse social contexts (Pollak 2012; Smith and Pollak 2021). Findings suggest that mother–child RSA coregulation is related to concurrent maternal behavioral support (Skoranski et al. 2017) and can reflect maternal buffering of children's physiological stress response (Lunkenheimer et al. 2019). However, there is limited understanding of these dyadic physiological responses among higher‐risk families with infants, especially when also considering differences in infant developmental status that could influence how much maternal support is needed. To deepen our understanding of how these vital physiological and developmental processes influence infants’ regulatory development in families with experienced adversity, namely those with lower income, higher stress, and child maltreatment risk, we investigate the relationship between dynamic trajectories of mother–infant RSA coregulation and infant developmental status in response to experimental variations in levels of maternal support and engagement. This research aims to identify potential intervention targets that can promote resilience or adaptation in vulnerable mother–infant populations.

### 1.1 Mother–Infant RSA Coregulation

RSA is a key component of the stress response system, helping to maintain or restore physiological homeostasis after a stressor is experienced (Beauchaine and Thayer 2015). Specifically, RSA is characterized by a rhythmic increase and decrease in heart rate within respiration cycles (Porges 2007). During non‐stressful conditions, higher RSA or increases in RSA (i.e., augmentation) contribute to a slowing down of heart rate and reflect adaptation to the environment (Graziano and Derefinko 2013), whereas lower RSA or decreases in RSA (i.e., withdrawal) during rest may heighten vulnerability to stress and are linked to the development of emotion dysregulation and psychopathology (Hinnant and El‐Sheikh 2009). However, during emotionally arousing and stressful conditions, RSA decreases signify a release of the “vagal brake,” thereby removing the inhibitory influence of the parasympathetic nervous system that maintains rest and homeostatic processes to allow mobilization of the sympathetic nervous system resources to meet a social challenge or threat (Berntson et al. 1991; Porges 2001). In contrast, RSA augmentation signifies a lack of regulatory effort, engagement, and/or avoidance or disengagement from a stimulus, which may be theoretically maladaptive in the context of social challenge or threat (Porges 2007; Thayer et al. 2009).

As operationalized in this study, dynamic trajectories of RSA coregulation refer to the mutual coordination and moment‐to‐moment fluctuations in mother–infant physiological responses, assessed across successive 30‐s epochs. The parasympathetic nervous system, indexed by RSA, plays a particularly important role in this process and is critical for supporting child development (Leerkes et al. 2017; Qu and Leerkes 2018; Whedon et al. 2018; Zeytinoglu et al. 2022). In the early months of life, mothers drive physiological exchanges with their infant. By 6 months of age, mother–infant coregulation becomes an essential component of early interactions, and the coordination of parasympathetic functioning between mothers and infants is central to the development of infants’ regulatory capacities, particularly their biological and behavioral responses to stress (Feldman 2007a). For example, recent evidence suggests that mother–infant coregulation of RSA during the first year is associated with later emotion regulation (Abney et al. 2021) and internalizing behaviors (Lan et al. 2024).

Studies examining time‐varying changes in maternal and child RSA have explored both concurrent and lagged coregulation to assess mutual physiological influence. Evidence suggests that mothers and children often exhibit concurrent RSA concordance (Lunkenheimer et al. 2015) and can coordinate heart rhythms within less than a 1‐s lag, particularly during moments of behavioral synchrony (Feldman et al. 2011). In high‐risk caregiving environments, RSA concordance may be disrupted; for example, Brown et al. (2021) found that lower maternal RSA predicted lower infant RSA across a stress‐induced paradigm, suggesting a pattern of interrelated physiological distress between mother and infant. Although RSA is recognized as a transdiagnostic marker of risk (Beauchaine and Thayer 2015; Brown et al. 2021), we still know relatively little about mother–infant RSA coregulation in relation to different caregiving contexts and infant developmental status, such as socioemotional skills, adaptive behaviors, and developmental milestones. The current study focused on concurrent RSA associations to address this gap, offering insight into how physiological coregulation may reflect or support infant adaptation among families experiencing adversity.

### 1.2 Caregiving Contexts and Mother–Infant Coregulation

Patterns of physiologically indexed regulation between mothers and infants may vary across caregiving contexts that differ in maternal support and engagement. During infancy, mothers play a central role in responding to their infants’ cues in ways that are both developmentally appropriate and shaped by the level of support they can provide. According to the theory of domain‐specificity of parenting (Grusec and Davidov 2010), different interaction contexts, such as those characterized by warmth, reciprocity, or joint social involvement, activate specific domains that influence caregiving behaviors as well as children's responses to these behaviors. In supportive contexts, maternal warmth and engagement tend to activate the domain of reciprocity, promoting mother–infant interactions that accommodate each other's needs as well as the development of children's regulatory skills (Jennings et al. 2008). In contrast, in more challenging caregiving contexts, maternal capacity to engage and support may be constrained, which can disrupt and limit reciprocity between mother and infant and potentially alter the typical course of coregulatory processes.

The Face‐to‐Face Still‐Face (FFSF) paradigm (Tronick et al. 1978) is one task commonly used to assess how mother–infant dyads behaviorally and physiologically respond to different caregiving contexts, specifically during alternating episodes of social engagement (i.e., play episodes) and disengagement (i.e., still‐face stressor or challenge episodes). Studies examining normative patterns of mothers’ individual physiological responses during the FFSF paradigm have found that mothers’ RSA increases during initial play and either remains the same or increases during the stressor episode, and then decreases during the re‐engagement episode (Moore et al. 2009; Oppenheimer et al. 2013). This is consistent with the assumption that mothers may be attempting to regulate or actively cope as they re‐engage with their infant following a stressor. For infants, normative patterns of individual RSA generally decrease during the stressor episode relative to the engagement/re‐engagement episodes (Conradt and Ablow 2010; Moore and Calkins 2004), suggesting infant mobilization of sympathetic nervous system resources to meet the challenge of maternal disengagement. Indeed, infants of mothers who are warmer and more responsive during engagement episodes may better adapt to the transition to stressful contexts (Haley and Stansbury 2003). However, not all infants show this expected RSA withdrawal; for example, Conradt and Ablow (2010) found that some infants show no RSA withdrawal when disengaged from their mother (Conradt and Ablow 2010). Furthermore, Moore and Calkins (2004) found that infants who do not demonstrate decreases in RSA during maternal disengagement often show lower dyadic coordination in play episodes with their mothers.

Recent modifications of the FFSF paradigm, such as the double FFSF (Haley and Stansbury 2003), have aimed to increase the level of infant distress by introducing a second stressor and re‐engagement episode. Research shows that infants often exhibit more negative responses during the second stressor compared to the first (Bosquet Enlow et al. 2011; DiCorcia et al. 2016; Haley 2011; Haley and Stansbury 2003). However, consistent with findings from the original FFSF, some studies have not observed heightened negative responses to repeated stressors (Montirosso et al. 2010; Tollenaar et al. 2011). Notably, this literature has largely overlooked maternal physiological responses during repeated stressor episodes, even though mothers are the primary regulators of dyadic interactions during infancy. Furthermore, no prior studies have examined mother–infant RSA coregulation within the double FFSF paradigm among families with lower income, higher stress, and child maltreatment risk, leaving a gap in understanding how these dyads respond during different caregiving contexts.

In studies of dyadic RSA coregulation during free play or other social engagement tasks, broadly, typical mother–infant dyads show robust physiological coordination. For example, while Feldman et al. (2011) found mothers and their 3‐month‐old infants coordinated their heart rhythms within less than 1‐s during social engagement, others have found mother–infant dyads show positive respiratory coregulation within 5‐s lags in a sample of older infants who ranged in age from 7 to 8.5 months (McFarland et al. 2020). These studies suggest that in low‐stress contexts, mothers and infants tend to match physiological states. However, during re‐engagement after a stressor, RSA patterns may diverge. For example, Ostlund et al. (2017) found maternal RSA withdrawal corresponded to time‐matched infant RSA augmentation, possibly reflecting an adaptive process in which the mother served as a stress buffer while their infant attempted to self‐regulate.

Together, these findings highlight that patterns of mother–infant physiological coregulation are not uniform but rather vary based on the caregiving context and the level of maternal support and engagement. Under supportive conditions, mothers’ engagement and regulatory efforts may scaffold infant self‐regulation. In more challenging contexts, coregulation may be disrupted as infants adapt their physiological responses to maternal availability. Understanding this variability is important to identifying typical as well as potentially maladaptive or adaptive coregulatory processes in families experiencing adversity.

### 1.3 Adversity and Mother–Infant RSA Coregulation

Adverse caregiving environments may influence early mother–infant coregulation. For instance, among mothers with higher maternal depressive symptoms, mother–child dyads show weakened concordance (McKillop and Connell 2018), greater discordance (Amole et al. 2017; Suveg et al. 2019; Woody et al. 2016), or no concordance in RSA (Lunkenheimer, Tiberio, et al. 2018). In addition, mothers at higher risk of maltreatment are less likely to show physiological coordination with their child than mothers without such risk (Skowron et al. 2010). Studies have found that maltreating mother–child dyads show either weakened or absent RSA concordance during dyadic tasks (Brown et al. 2021; Creaven et al. 2014; Giuliano et al. 2015; Lunkenheimer, Busuito, et al. 2018; Skowron et al. 2013) compared to non‐maltreating dyads. Lunkenheimer, Busuito, et al. (2018) found that non‐maltreating mothers and their preschool‐aged children showed positive RSA concordance, while dyads characterized by abuse showed weaker concordance and those characterized by neglect showed none. Specific to infancy, Brown et al. (2021) found that greater severity of child maltreatment risk is associated with weakened RSA concordance in mother–infant dyads during the FFSF. These findings suggest that mothers at increased risk of maltreatment and other adversity may struggle to regulate in stressful caregiving contexts, leading to reduced attunement to their infant's needs and altered mother–infant RSA coregulation.

Further, a meta‐analysis of RSA responses during the FFSF paradigm found that high‐risk infants showed the typical RSA decreases during the stressor episode but did not show the normative increase in RSA during re‐engagement (Jones‐Mason et al. 2018). However, when mothers demonstrate higher levels of support and repair in challenging mother–child interactions, children's RSA reactivity may be buffered, even among high‐risk families (Lunkenheimer et al. 2019). In these cases, the mother's active engagement, typically reflected in their moderate RSA withdrawal, may help the child become less distressed, providing a protective effect during a challenging interaction. Nonetheless, it remains unclear to what extent these coregulatory processes promote infant resilience or adaptation across different maternal caregiving contexts within adversity‐exposed dyads, or whether their effects are shaped by the infant's developmental status.

### 1.4 Developmental Status and Mother–Infant Coregulation

Early in life, infants largely rely on their mothers as external regulators (Feldman 2007b). When mothers respond to infants’ cues in ways that meet their needs, these interactions foster a sense of safety and security (Bowlby 1982) and lay the foundation for developing self‐regulatory capacities (Feldman 2007b). According to dynamic systems theory, the mother–infant dyad operates as a dynamic system that self‐organizes into predictable emotional, behavioral, and physiological patterns that serve a function for the system (Lunkenheimer et al. 2011). When these patterns are consistent and well‐coordinated, such as through synchronized affect or behavior, they support the infant's emerging ability to self‐regulate (Feldman 2007b). Indeed, there is strong empirical support linking parent–child behavioral self‐regulation and coregulation across caregiving contexts to later developmental outcomes (e.g., Braungart‐Rieker et al. 2001; Cohn et al. 1991; Ekas et al. 2012; Lobo and Lunkenheimer 2020; Lunkenheimer et al. 2020; Mesman et al. 2009; Moore et al. 2001), suggesting that the organization of early behavioral exchanges may serve as markers of infant development and self‐regulatory capacity.

Building on this behavioral work, theory and research highlight the role of physiological processes, such as mother–infant RSA coregulation, as fundamental to infant self‐regulation and broader aspects of developmental functioning. Consistent with polyvagal theory (Porges 2001), physiological regulation reflects the neurobiological infrastructure that supports social engagement and adaptive functioning. During infancy, caregivers scaffold the infant's developing physiological system (Feldman 2007b). Prior research shows that infant RSA tends to become more organized with age, such that older infants generally show higher baseline RSA and more consistent RSA withdrawal in response to challenge (Doussard‐Roosevelt et al. 1997; Porges and Furman 2011). Furthermore, greater RSA maturation, reflected in higher RSA during social play and adaptive RSA withdrawal during stress, has been strongly linked to more advanced social competence (i.e., the ability to form positive relationships and interact effectively with caregivers and others), as well as enhanced cognitive processing and gross motor abilities (Bradshaw and Abney 2021; daSilva and Bertenthal 2025; Doussard‐Roosevelt et al. 1997), thereby supporting the role of RSA as an important developmental marker. In addition, a systematic review of RSA coregulation (DePasquale 2020) found that positive caregiver–child interactions and better socioemotional skills across childhood are associated with parent–child RSA coregulation. However, findings are not uniform across all samples, and research is lacking with infants. Some work has shown no association between RSA coregulation and parent–child interaction quality or child functioning in families experiencing high psychosocial and parental risk (Suveg et al. 2016). Thus, it is possible that for families with experienced adversity, certain patterns of RSA coregulation may depend on the broader caregiving environment, including the degree of maternal support and engagement, as well as on infants’ developmental capacities. From a transactional model of development perspective (Sameroff 2009), infants with lower social competence or delays in reaching developmental milestones may experience difficulty regulating in dyadic contexts, reflected in less coordinated trajectories of maternal–infant RSA. It is also possible that mothers may need to exert greater effort and responsiveness to support infants with poorer developmental functioning (Feldman 2007b). Conversely, when infants have stronger developmental skills and abilities, it may lessen the pressure on mothers to provide more support and engagement, thus allowing better physiological coordination and less stressful interactions (R. A. Thompson 2014).

Despite extensive recognition of the importance of physiological processes in early development, empirical evidence linking mother–infant RSA coregulation directly to the infant's developmental status remains scarce, and most studies have focused on individual‐level RSA trajectories rather than dyadic coordination. Moreover, this literature has primarily focused on infants’ social competence and communicative ability, with less attention to broader adaptive functions. We know that behaviors reflecting a child's functional independence serve as regulators of physiological states (Porges and Furman 2011), yet their contribution to early mother–infant coregulation remains underexplored. The current study extends this literature by examining mother–infant RSA coregulation during the double FFSF in relation to a broader set of developmental domains, including socioemotional skills and general developmental milestones (e.g., cognition, language), as well as adaptive behavior (e.g., self‐feeding, self‐help behaviors). Understanding these nuanced associations may clarify how individual differences in infant development shape early physiological regulatory processes, thereby informing interventions that promote positive mother–infant interactions and infant development, particularly in families experiencing adversity.

### 1.5 The Current Study

The extant research on dyadic physiological coregulation (also synchrony) is limited because we know relatively little about the developmental correlates of RSA coregulation in infancy, and few studies situate mother–infant coregulation findings in relation to differences in caregiving contexts that vary in maternal support and engagement, which may be important to inform intervention. Given that RSA is a transdiagnostic marker of risk, it is thus critical to examine mother–infant RSA coregulation in samples with experienced adversity, such as those with lower income, higher stress, and child maltreatment risk, to better understand whether such a target might promote adaptive parenting and infant development. Therefore, the overall objective of the current study was to examine whether dynamic trajectories of mother–infant RSA coregulation were associated with infant socioemotional skills, adaptive behavior, and developmental milestones in conditions that varied in maternal support and engagement. Given the complexity and limited research on RSA coregulation patterns in high‐risk mother–infant dyads, we hypothesized that distinct patterns of coregulation may emerge among families experiencing adversity. Specifically, weaker or discordant RSA coregulation may not uniformly be linked to poorer infant developmental outcomes and, in some caregiving contexts, could be associated with better infant functioning.

## 2 Materials and Methods

### 2.1 Participants

Participants were 53 mother–infant dyads from a larger longitudinal study oversampled for lower income, higher stress, and child maltreatment risk. Families were recruited through departments of human services and public health and assessed at baseline when infants were approximately 6–14 months old (*M* = 8.30, SD = 2.49). This age range captures a key developmental period characterized by rapid growth in social engagement, physiological regulation, and emerging socioemotional skills. While many mother–infant interaction task studies focus on younger infants, others have examined broader age ranges (Stockdale et al. 2020; Weinberg et al. 2012). Our aim was to assess mother–infant RSA coregulation across this transitional window to explore differences in physiological coregulation. We included English‐ and Spanish‐speaking families who were screened for receipt of financial assistance (84.9%), involvement with child protective services (52.8%), or experiencing other life stressors (e.g., family conflict: 26.4%; parental separation: 35.8%). Participants resided in the rocky mountain region of the United States. All mothers identified as female and were on average 29.74 years old (SD = 6.71). They racially and ethnically identified as White non‐Latina (47.2%), followed by Latina (22.6%), Black/African American (11.3%), multi‐race (7.5%), American Indian (5.7%), Asian (3.8%), and unknown (1.9%). At baseline, 56.6% of mothers were married or had a domestic partner, 34.0% were single, and 9.4% were separated, widowed, or unknown. Regarding employment status, 41.5% were unemployed, 28.3% employed, 18.9% stayed home with their child, and 11.3% were students or had unknown employment. Slightly less than half (45.3%) of mothers had a high school education or below, followed by some college (32.1%), a 4‐year college degree (15.1%), or a postgraduate degree (7.5%). Infants were 50.9% male and 49.1% female. In addition, their race and ethnicity was 34.0% White non‐Latino, 24.5% Latino, 24.5% multi‐race, 11.3% Black/African American, 1.9% American Indian, 1.9% Asian, and 1.9% unknown. On average, there were approximately two children (SD = 1.28) in the household.

### 2.2 Procedure

For the current study, mother–infant dyads participated in two home‐based visits over a 1‐week period. During the first visit, mothers completed informed consent and self‐report questionnaires, and infants completed developmental assessments. During the second visit, mother–infant dyads participated in a 12‐min double FFSF paradigm that consisted of six conditions, each approximately 2 min in duration: (1) mothers and infants were seated across from each other for baseline; (2) mothers were instructed to play with their infant like normal (an initial free play episode); (3) mothers were asked to withdraw play and keep a still, expressionless face and refrain from touching their infant (still face stressor episode); (4) mothers were instructed to play with their infant like normal (a recovery/re‐engagement play episode); (5) still face stressor episode was repeated; and (6) recovery/re‐engagement play episode was repeated. Assessments of both the mothers’ and infants’ RSA were measured in 30‐s epochs across the entire paradigm (i.e., approximately four assessments per condition). The use of the double FFSF builds on prior research suggesting that some infants show heightened negative responses while others show adaptation during a second stressor episode (e.g., Haley and Stansbury 2003), potentially providing a more sensitive test of coregulatory processes. If the infant became fussy for more than 20‐s during the paradigm, the interaction was stopped, and the infant was soothed by their mother. Moreover, families were excluded from the study if infants had a chronic illness or developmental disorder that would interfere with physiological assessment. All procedures for this study were approved by the University Institutional Review Board.

### 2.3 Measures

#### 2.3.1 Adaptive Behavior

Infant adaptive behavior was assessed using standard scores on the Developmental Assessment for Young Children‐2 Adaptive Behavior subscale (DAYC‐2‐AB; Voress and Maddox 2013). The DAYC‐2‐AB measures independent self‐help functioning, such as feeding, dressing, and taking personal responsibility. For each domain of the DAYC‐2, the entry point (first item scored) is determined by the infant's chronological age. Items were scored as “passed” = 1 or “not passed” = 0 and examined through observation, direct assessment, and caregiver interview. Items were summed until three consecutive items received a score of 0 (i.e., to establish a ceiling) to create raw scores. Raw scores were then converted to standard scores for final analysis. In the current sample, standard scores on the DAYC‐2‐AB ranged from 65 to 114 (*M* = 91.98, SD = 9.50).

#### 2.3.2 Socioemotional Skills

Similar to infant adaptive behavior, infant socioemotional skills were assessed using standard scores on the Developmental Assessment for Young Children‐2 Social‐Emotional subscale (DAYC‐2‐SE; Voress and Maddox 2013). The DAYC‐2‐SE measures social awareness, social competence, and social relationships with parents, caregivers, peers, and others in their environment. For each domain of the DAYC‐2, the entry point (first item scored) is determined by the infant's chronological age. Items were scored as “passed” = 1 or “not passed” = 0 and examined through observation, direct assessment, and caregiver interview. Items were summed until three consecutive items received a score of 0 (i.e., to establish a ceiling) to create raw scores. Raw scores were then converted to standard scores for final analysis. In the current sample, standard scores on the DAYC‐2‐SE ranged from 74 to 115 (*M* = 92.75, SD = 9.19).

#### 2.3.3 Developmental Milestones

Infant developmental milestones were assessed using total scores on the Survey of Well‐Being of Young Children Developmental Milestones subscale (SWYC‐DM; Perrin et al. 2016). The 10‐item SWYC‐DM measures infant's cognitive, language, and motor development. Caregivers reported the extent to which their infant is doing each item (0 = *not yet*, 1 = *somewhat*, 2 = *very much*). Items were summed to create a total score. In the current sample, total scores on the SWYC‐DM ranged from 4 to 20 (*M* = 14.38, SD = 3.51).

#### 2.3.4 Respiratory Sinus Arrhythmia

During the mother–infant interaction paradigm, mother and infant cardiac physiology was measured with the Bittium Faros Sensor 180, which is a portable, wearable, and externally applied electrocardiograph (ECG) recorder and wireless transmitter for ECG measurement and R‐R interval data measurement. Three disposable wet gelled pediatric and adult electrodes were placed in a 3‐lead position with one channel on the distal ends of the right and left clavicle and lower left abdomen for infants and mothers, respectively. Heart rate data were measured with an ECG sampling frequency of 1000 Hz.

Three programs were used to synchronize, edit, and analyze the ECG data for cardiac rhythms. First, CardioPeak and Segmenter (Brain‐Body Center for Psychophysiology and Bioengineering 2019a) were used to extract the R–R peaks from the ECG data and segment the data by condition. Second, CardioEdit software (Brain‐Body Center 2007) was used to review and edit the resulting heart period (IBI) files for artifacts. Editing consisted of adjusting incorrect R‐peak identifications and/or integer arithmetic (i.e., dividing intervals between heartbeats when detections of R‐peaks from the ECG were missed or adding intervals when spuriously invalid detections occurred). Third, CardioBatch Plus Synchrony software (Brain‐Body Center for Psychophysiology and Bioengineering 2019b) was used to analyze the synchronized edited IBI files between mother–infant dyads. RSA was calculated using the Porges–Bohrer method (Porges 1985), which quantifies the amplitude of RSA with age‐specific parameters that are sensitive to the maturational shifts in the frequency of spontaneous breathing.

The Porges–Bohrer method, as applied in CardioBatch Plus Synchrony, includes the following steps: (1) R–R intervals are timed to the nearest ms to produce a time series of sequential heart periods; (2) sequential heart periods are both resampled to 5 Hz to maintain synchrony within the dyad when producing time‐based data; (3) the time‐based series is detrended by a cubic moving polynomial (53‐point for adults; 21‐point for infants) (Porges and Bohrer 1990) that is stepped through the data to create a smoothed template, and the template is subtracted from the original time‐based series to generate a detrended residual series; (4) the detrended time series is bandpassed to extract the variance in the heart period pattern associated with spontaneous breathing (0.12–0.40 Hz for adults; 0.3–1.3 Hz for infants); and (5) the natural logarithm of the variance of the bandpassed time series is calculated as the measure of the amplitude of RSA (Riniolo and Porges 1997). These procedures are statistically equivalent to frequency domain methods (i.e., spectral analysis) for the calculation of the amplitude of RSA when heart period data are stationary (Porges and Byrne 1992). RSA and heart period were quantified during each sequential 30‐s epoch within each 2‐min condition of the mother–infant interaction paradigm (i.e., four completed 30‐s epochs in each 2‐min condition for a total of 20 possible epochs during the dyadic interaction were used in analysis).

### 2.4 Data Analysis

Descriptive statistics were calculated for mother and infant sample characteristics. Exploratory linear mixed models were conducted to examine mother and infant RSA patterns by developmental status across task conditions. Models specifying the effects of mother‐on‐infant and infant‐on‐mother RSA coregulation were performed separately (e.g., to examine how infants physiologically respond to their mothers as a function of their developmental status). Linear mixed models were fit using the R lmer function from the lme4 package (Bates et al. 2015), version 1.1.29, and Satterthwaite degrees of freedom implemented using the R lmerTest package (Kuznetsova et al. 2017), version 3.1.3, using R 4.2.0 (R Core Team 2021). To account for the clustered nature of the data (i.e., multiple observations within each dyad), all models contained a random intercept for dyad. Mixed models represent the intent‐to‐treat (ITT) approach in longitudinal data analysis as every time point that contains complete data is included in the models, including participants who do not complete all assessment points (Chakraborty and Gu 2009). To evaluate change across time and change in RSA coregulation between the play and stressor episodes, all conditions in the interaction paradigm other than the baseline condition were used in analyses. Of the 20 possible epochs in the five conditions used in the analyses, dyads completed an average of 18.20 (SD = 3.62) of the 20 possible epochs. Missing data were evaluated using Little's (1988) Missing Completely at Random test. Data were assumed to be missing at random.

Longitudinal models were constructed following recommendations from Singer and Willett (2003) in which an unconditional growth model (i.e., a model with only parameters for time) is first established, and variables of theoretical interest are added subsequently. The 20 total epochs were coded sequentially. We evaluated the following unconditional growth models to evaluate the nature of change in both infant RSA and mother RSA: (1) unconditional time (i.e., no time variables); (2) linear time; (3) quadratic time (i.e., linear and quadratic time variables); (4) natural log time; (5) stress versus play conditions, in which a dummy variable was coded 1 for Conditions 3 and 5 (still face stressor episodes) and was 0 otherwise; and (6) all conditions, in which a dummy variable was applied to Conditions 3 through 6 and Condition 2 served as the reference condition. The unconditional growth models thus represented a variety of possible models of change over time; the first four models above evaluated change across time irrespective of the experimental conditions, and the final two models evaluated time trends that were a function of the experimental setup (e.g., stress vs. play conditions).

Unconditional growth models were compared using the models’ Akaike information criterion (AIC) values. The best fitting unconditional growth model was determined by the lowest AIC value, and additionally, the best fitting model was required to be lower by 2 than the simplest model, the unconditional growth model. An AIC difference of 2 or greater is the criterion for a substantially better fitting model (Burnham and Anderson 2002). In addition to statistical fit, model selection was informed by alignment with observed individual trajectories of RSA across the paradigm.

After selecting the best unconditional growth model for infant RSA and mother RSA, separate coregulation models were constructed for the three measures of infant developmental status: adaptive behavior, socioemotional skills, and developmental milestones. Measures of infant development were grand mean‐centered so that the interpretation of parameters common to all models was comparable across models. Each coregulation model contained time parameters from the best fitting unconditional growth model, the other dyad member's RSA (e.g., the model in which infant RSA was the dependent variable contained mother RSA as an independent variable), and a measure of infant development. All possible interactions between these three variables were included in the model.

## 3 Results

### 3.1 Individual Infant and Mother RSA Models

**FIGURE 1:**
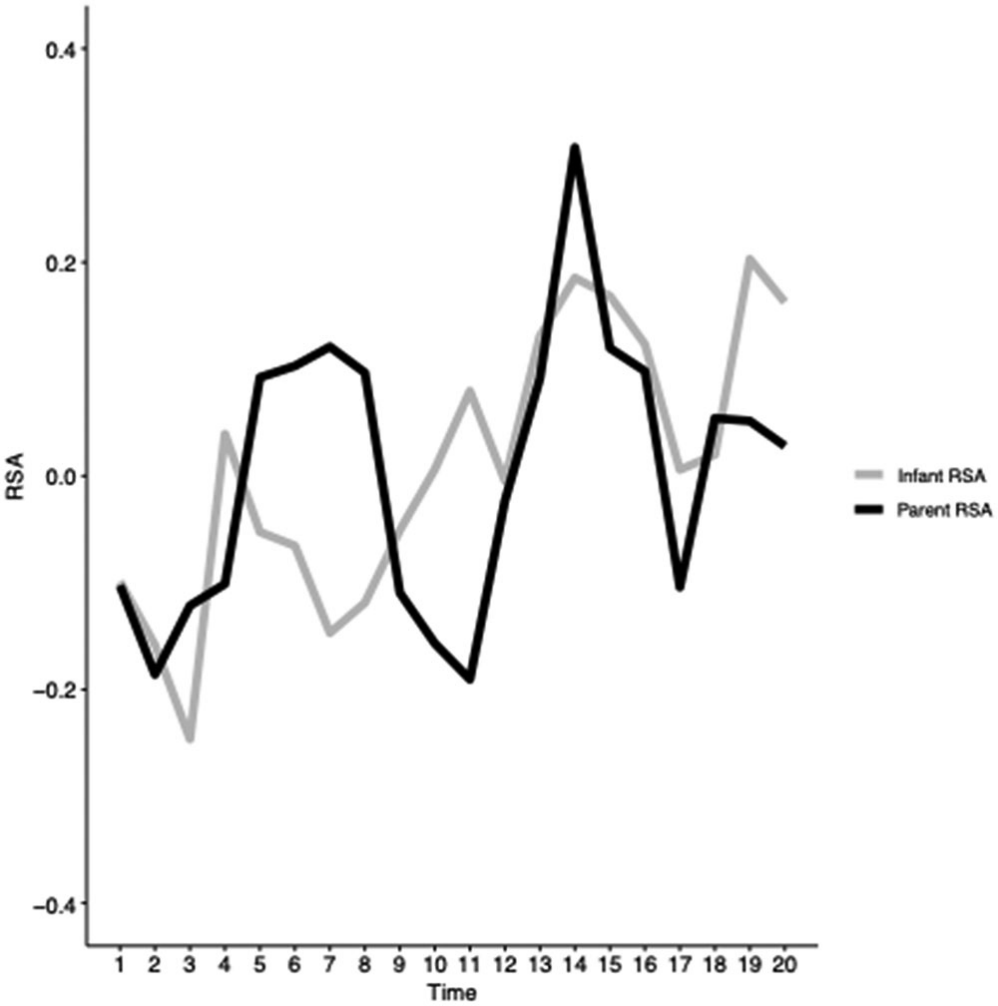
Individual RSA trajectories for infants and mothers across the paradigm. The time values correspond to sequential 30‐s epochs (total duration = 10 min).

Figure 1 illustrates individual RSA trajectories for infants and mothers across the FFSF paradigm. Overall, mothers showed RSA augmentation during both stressor episodes and RSA withdrawal during play episodes. In contrast, infants showed RSA withdrawal during the initial stressor episode, followed by a nonlinear increase in RSA across subsequent episodes.

Step one of data analysis examined growth models for individual infant RSA and mother RSA over time and across task conditions. Log‐transformed time was the best fitting unconditional growth model for infant RSA. Log‐transformed time was a positive significant predictor of infant RSA (*t*[916] = 3.06, *p* = 0.002). The fact that the log‐transformed time was a better fit than the linear time model indicated that the change in infant RSA tapered off over the duration of the task paradigm as opposed to a linear trend, which models a constant change. In contrast, with respect to mother RSA, the stress versus play model was the best fitting unconditional growth model. Mother RSA was significantly higher in stress conditions relative to play conditions (*t*[910] = 4.59, *p* < 0.001). See Table 1 for fit indices for the unconditional growth models.

**TABLE 1:** Akaike information criterion (AIC) fit indices for unconditional growth models.

	Outcome	
Model	Mother's RSA	Infant's RSA
Unconditional	2329.54	2612.76
Linear	2336.00	2611.96
Quadratic	2349.34	2625.98
Log	2330.96	2610.30
Stress (vs. play)	2314.96	2618.25
All conditions	2327.00	2620.30

### 3.2 Mother–Infant and Infant–Mother RSA Coregulation Models

**FIGURE 2:**
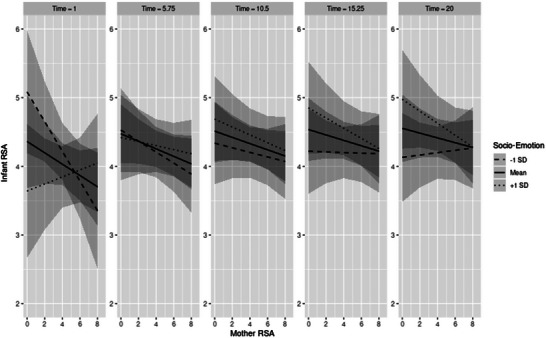
Simple slopes for infant RSA estimated from the log of time × mother RSA × socioemotional skills model. Panels represent estimates at Time 1 (1st percentile, initial free play episode), 5.75 (25th percentile, initial still face stressor episode), 10.5 (50th percentile, initial recovery play episode), 15.25 (75th percentile, final still face stressor episode), and 20 (100th percentile, final recovery play episode). Separate lines represent slopes of infant RSA regressed on socioemotional skills at 1 SD below the mean, the mean, and 1 SD above the mean.

Step two of data analysis examined mother‐on‐infant and infant‐on‐mother RSA coregulation effects as a function of time and infant development. The three‐way interaction between time, mother RSA, and adaptive behavior, as well as the three‐way interaction between time, mother RSA, and developmental milestones, was not a significant predictor of infant RSA. However, as shown in Table 2, the three‐way interaction between time, mother RSA, and infant socioemotional skills was significant (*t*[912] = −2.56, *p* = 0.011). Results for the model‐predicted infant RSA values are plotted in Figure 2. When infants had higher socioemotional skills, mother–infant dyads showed more coordinated RSA at the start of the task paradigm but became less coordinated as the paradigm progressed. Specifically, when mother RSA was lower, infant RSA was higher, and this relation became more pronounced over time, suggesting that maternal engagement (RSA withdrawal) buffered infant stress (RSA augmentation) throughout the paradigm. In contrast, among infants with lower socioemotional skills, mother–infant dyads showed less coordination in their RSA at the beginning of the paradigm that weakened over time, such that there was no relation between infant RSA and mother RSA by the end of the task paradigm. Results of the two‐way interaction between mother RSA and infant socioemotional skills predicting infant RSA also indicated that, overall, the effects of individual differences in infants’ socioemotional skills were more pronounced when mothers showed lower RSA on average, indicating their greater engagement and arousal during the task paradigm, but minimal when mothers showed higher RSA, especially by the end of the task (e.g., mother RSA augmentation).

**TABLE 2:** Infant RSA regressed on mother RSA, the log of time, and measures of infant development.

Outcome	Parameter	*B*	SE	*t*	df	*p*
Adaptive Behavior	Intercept	4.76	0.35	13.69	705	< 0.001***
	Mother RSA	−0.15	0.07	−2.19	931	0.029*
	Log of time	−0.10	0.13	−0.79	892	0.427
	Adaptive Behavior	−0.02	0.03	−0.74	558	0.459
	Mother RSA × Log of time	0.05	0.03	1.69	892	0.091
	Mother RSA × Adaptive Behavior	0.00	0.01	0.64	935	0.525
	Log of time × Adaptive Behavior	0.02	0.01	1.55	892	0.122
	Mother RSA × Log of time × Adaptive Behavior	0.00	0.00	−1.16	890	0.248
Socioemotional Skills	Intercept	4.38	0.34	12.71	717	< 0.001***
	Mother RSA	−0.09	0.07	−1.23	952	0.218
	Log of time	0.06	0.13	0.42	913	0.676
	Socioemotional Skills	−0.08	0.04	−2.19	627	0.029*
	Mother RSA × Log of time	0.02	0.03	0.62	915	0.538
	Mother RSA × Socioemotional Skills	0.01	0.01	2.05	951	0.041*
	Log of time × Socioemotional Skills	0.04	0.01	3.31	909	0.001**
	Mother RSA × Log of time × Socioemotional Skills	−0.01	0.00	−2.56	912	0.011*
Developmental Milestones	Intercept	4.68	0.35	13.38	660	< 0.001***
	Mother RSA	−0.14	0.07	−1.98	890	0.048*
	Log of time	−0.09	0.13	−0.66	853	0.507
	Developmental Milestones	0.09	0.10	0.89	679	0.372
	Mother RSA × Log of time	0.04	0.03	1.43	856	0.153
	Mother RSA × Developmental Milestones	−0.01	0.02	−0.35	881	0.730
	Log of time × Developmental Milestones	−0.03	0.04	−0.75	856	0.453
	Mother RSA × Log of time × Developmental milestones	0.00	0.01	0.13	861	0.896

*Note:* Models are identical with the exception of the measures of infant development.

*p < 0.05.

**p < 0.01.

***p < 0.001.

**FIGURE 3:**
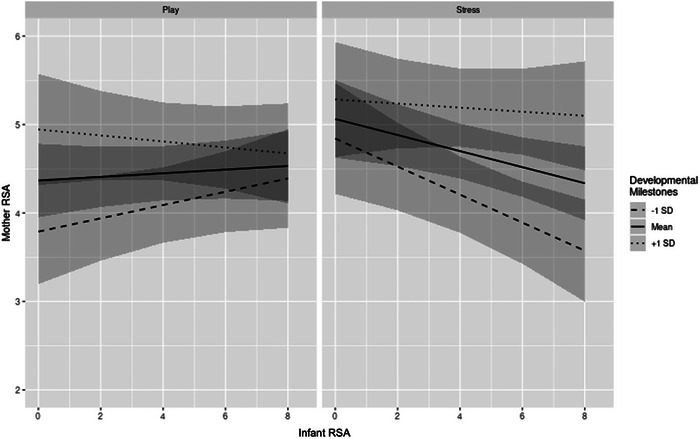
Simple slopes for mother RSA estimated from the stress versus play condition × infant RSA × developmental milestones interaction model. Panels represent estimates for play and stress conditions. Separate lines represent slopes of infant RSA regressed on developmental milestones at 1 SD below the mean, the mean, and 1 SD above the mean.

The three‐way interaction between stress versus play condition, infant RSA, and adaptive behavior, and the three‐way interaction between stress versus play condition, infant RSA, and socioemotional skills were not significant predictors of mother RSA. However, as shown in Table 3, the three‐way interaction between stress versus play condition, infant RSA, and developmental milestones was significant (*t*[856] = 2.64, *p* = 0.008). Results for the model‐predicted mother RSA values are plotted in Figure 3. These findings demonstrated that among infants with lower developmental milestones, mother–infant dyads showed more coordination in their RSA during the play condition on average, evidenced by lower mother and infant RSA, suggesting that play may be more effortful or challenging for dyads when infants have fewer resources. Additionally, during the stress condition, higher infant RSA was associated with lower mother RSA for dyads with lower infant developmental milestones, suggesting that mothers’ stress response was activated when infants were less engaged during the still‐face stressor. In contrast, among infants with higher developmental milestones, there was no significant difference in RSA coregulation across the play and stress conditions.

**TABLE 3:** Mother RSA regressed on infant RSA, stress versus play condition, and measures of infant development.

Outcome	Parameter	*B*	SE	*t*	df	*p*
Adaptive Behavior	Intercept	4.53	0.21	21.54	147	< 0.001***
	Infant RSA	0.00	0.03	0.03	917	0.977
	Stress vs. Play Condition	0.56	0.17	3.26	889	0.001**
	Adaptive Behavior	0.00	0.02	0.09	143	0.931
	Infant RSA × Stress vs. Play Condition	−0.09	0.04	−2.24	889	0.025*
	Infant RSA × Adaptive Behavior	0.00	0.00	−0.55	914	0.583
	Stress vs. Play Condition × Adaptive Behavior	0.01	0.02	0.34	888	0.735
	Infant RSA × Stress vs. Play Condition × Adaptive Behavior	0.00	0.00	−0.06	888	0.952
Socioemotional Skills	Intercept	4.38	0.21	21.30	157	< 0.001***
	Infant RSA	0.03	0.03	0.78	939	0.434
	Stress vs. Play Condition	0.76	0.17	4.44	908	< 0.001***
	Socioemotional Skills	0.02	0.02	1.03	155	0.303
	Infant RSA × Stress vs. Play Condition	−0.13	0.04	−3.32	909	0.001**
	Infant RSA × Socioemotional Skills	0.00	0.00	0.16	936	0.871
	Stress vs. Play Condition × Socioemotional Skills	0.01	0.02	0.55	907	0.586
	Infant RSA × Stress vs. Play Condition × Socioemotional Skills	0.00	0.00	0.55	907	0.584
Developmental Milestones	Intercept	4.35	0.21	20.59	153	< 0.001***
	Infant RSA	0.02	0.03	0.67	883	0.506
	Stress vs. Play Condition	0.71	0.18	3.97	852	< 0.001***
	Developmental Milestones	0.16	0.06	2.52	205	0.012*
	Infant RSA × Stress vs. Play Condition	−0.12	0.04	−2.82	853	0.005**
	Infant RSA × Developmental Milestones	−0.02	0.01	−1.45	884	0.147
	Stress vs. Play Condition × Developmental Milestones	−0.10	0.06	−1.73	855	0.084
	Infant RSA × Stress vs. Play Condition × Developmental Milestones	0.03	0.01	2.64	856	0.008**

*Note:* Models are identical with the exception of the measures of infant development.

*p < 0.05.

**p < 0.01.

***p < 0.001.

To evaluate the power of the models presented above, we conducted sensitivity power analyses using the effective sample size (ESS; i.e., the number of independent and identically distributed observations that provide the same information as clustered data) derived from the sample size (*N* = 53) with 20 repeated measures and the median observed intraclass correlation coefficient = 0.62, which resulted in an ESS = 83. For power = 0.80, a two‐tailed alpha = 0.05, and an ESS = 83, the models are powered to detect effect sizes as small as *r* = 0.30 (medium effect size). We estimated standardized parameters using the pseudo standardization method in which fixed effects are divided by the conditional standard deviation of the outcome, reflecting variability after accounting for random effects (Lüdecke et al. 2019). The median observed standardized parameter for significant parameters in our models is 0.36 (*r* = 0.35), which is consistent with a detectable effect size determined from the power analysis.

## 4 Discussion

To our knowledge, this is the first paper to link dynamic changes in mother–infant RSA coregulation to infant developmental status while considering the caregiving context in which maternal support and engagement play a theoretically critical role in fostering infants' developing physiological regulation. First, individual trajectories of maternal and infant RSA in our sample were generally consistent with prior research, though some differences emerged. In our sample, infant RSA decreased during the initial stressor episode, and maternal RSA increased, aligning with common patterns observed in the literature (e.g., Ritz et al. 2012; Swider‐Cios et al. 2024). Mothers in our study also showed RSA augmentation during the second stressor episode. However, although prior studies often report continued RSA withdrawal in infants during the second stressor episode (e.g., Ritz et al. 2012), this pattern was not observed in our data and diverges from previous research. Instead, baseline growth models predicting infant RSA showed that log‐transformed time best fit the data. This nonlinear trajectory suggests that infants showed RSA withdrawal during the initial stressor, followed by a gradual increase and stabilization in RSA as the paradigm progressed, possibly reflecting adaptation to the caregiving context. Among higher‐risk families, it is possible that this pattern may instead reflect reduced regulatory flexibility, as children in adverse environments show heightened physiological sensitivity to novel or challenging contexts but may lack the capacity to sustain physiological engagement over time (Conradt et al. 2014; Obradović et al. 2010; Suurland et al. 2018). In contrast, maternal RSA followed a more stable pattern across task conditions in our models, which may reflect mothers’ greater regulatory capacity or their role in buffering infant stress responses and helping them adapt more effectively to the second stressor episode (Skoranski et al. 2017).

Second, the findings revealed that trajectories of mother–infant RSA coregulation are associated with infant developmental status and that this relationship varies depending on the different caregiving contexts. Specifically, mothers showed RSA withdrawal, and infants showed RSA augmentation when infants had higher socioemotional skills. Mothers’ RSA withdrawal may serve as a buffer against infant stress responses during the interaction paradigm, such that mothers’ support may have been more effective or infants may have been better able to make use of this support due to the greater ease of social engagement or stronger attachment security associated with higher infant socioemotional competence (compared to infants with lower levels of these skills). When infants had lower reported developmental milestones, this same theoretically supportive dyadic RSA pattern of maternal RSA withdrawal combined with infant RSA augmentation was observed specifically during stress conditions, suggesting mothers supported children who needed more assistance during challenge. However, for infants with higher developmental milestones, there was no significant difference in RSA coregulation between the play and stress conditions.

Regarding infant socioemotional skills, variations in mother–infant RSA coregulation were found during the paradigm. Infants with higher socioemotional competence demonstrated more adaptive stress regulation. Mothers appeared to activate their stress response systems (indicated by RSA withdrawal) during repeated stressors and re‐engagement episodes, which may support infants in regulating stress. This coordination between mother and infant may be more adaptive among infants with higher socioemotional competence because they may be more responsive to their mother's engagement efforts or more skilled/effective in seeking support when they need it. These findings are consistent with prior FFSF research and suggest that the paradigm is functioning as intended (Busuito et al. 2019). Additionally, they align with polyvagal theory (Porges 2007), which posits that autonomic system functioning is shaped by both internal states (e.g., stress) and external social interactions, especially in challenging contexts. In contrast, dyads with infants who had lower socioemotional skills exhibited a tapering off of RSA coregulation by the end of the paradigm, particularly when mothers were instructed to disengage. This suggests that lower socioemotional skills could contribute to challenges in coordinating RSA responses between mothers and infants. Moreover, the lack of association between mother RSA and infant RSA in some dyads with lower socioemotional skills may indicate impaired coping and regulatory abilities (e.g., insufficient maternal engagement or support), highlighting a potential target for future intervention efforts.

Mother–infant RSA coregulation also varied based on infant developmental milestones. For infants with higher developmental milestones, there was no significant difference in RSA coregulation between the play and stress conditions. This could suggest that mothers of more developmentally competent infants do not need to engage in as much physiological regulation because they recognize that their infants are better equipped to manage stress and participate in social interaction independently. This interpretation is supported by prior research indicating that when infants demonstrate strong developmental skills, it can reduce the burden on mothers to provide intensive support and engagement (R. A. Thompson 2014). In contrast, among infants with lower developmental milestones, mother–infant dyads showed physiological coordination during play conditions, with both showing RSA withdrawal. However, this coordination decreased during stress conditions, such that maternal RSA was lower when infant RSA was higher. Coordinated RSA withdrawal during play may reflect that typical social or play interactions are less frequent and require greater physiological effort in dyads with infants who have lower developmental milestones, compared to when the dyad is disengaged from one another. Additionally, in families exposed to adversity, typical social or play interactions may be more challenging for both mothers and infants. Consistent with prior research (Feldman 2007b), mothers may find it difficult to engage, while infants with lower developmental milestones may have difficulty participating due to limited developmental capacity or heightened sensitivity to the demands of the caregiving context. Under stressful conditions, these mothers may also find these situations more effortful but serve as a buffer of infant stress in an attempt to support their infant during challenging contexts.

It was unanticipated that the third infant outcome, adaptive behavior, would be unrelated to RSA coregulation in mother–infant dyads. One possibility is that these behaviors, reflecting daily living/self‐help functioning skills (e.g., feeding, dressing), are less central to moment‐to‐moment physiological coordination and may not serve as salient developmental indicators in dyadic interaction. Alternatively, though infant adaptive behaviors spanning cognitive, motor, social, and behavioral domains are crucial for overall child development, their influence on mother–infant RSA coregulation may be less important than that of socioemotional competence and developmental milestones, which more directly shape in‐the‐moment mother–infant interactions in contexts that vary in maternal support and engagement.

In summary, since RSA is a biomarker for stress regulation in social interactions (Porges 2007), it is postulated that differences in mother–infant RSA coregulation would be related to developmental status. Specifically, higher social competence in infants is likely to result in more adaptive coregulation between mothers and infants. However, developmental milestones encompass a broader range of skills beyond social competence. Delays in these milestones could influence how mothers perceive and respond to their infants (Paczkowski and Baker 2008), potentially altering the nature of their interactions. Mothers may need to exert more effort to support infants who require additional help with self‐regulation, which could lead to decreased coordination in RSA coregulation under challenging contexts. These differences highlight the importance of considering both social and nonsocial developmental factors when examining mother–infant RSA, especially among mother–infant dyads with experienced adversity. Future research would benefit from further testing the utility of tasks that elicit varying levels of maternal support and engagement, particularly among infants with differing levels of developmental proficiency.

### 4.1 Limitations

The current study is not without limitations. Although we used linear mixed modeling of physiological indices of regulation among mothers and infants to explore our research questions and strengthen analytic power, a larger sample could have allowed us to account for more sociodemographic and contextual factors that may interact with infant developmental status and mother–infant RSA coregulation. This study is a critical first step in evaluating infant developmental status in relation to dyadic RSA coregulation in mother–infant dyads with experienced adversity, yet there remain limitations with the measurement and analysis of RSA. Modeling RSA in mutually exclusive 30‐s epochs within‐phase of a specific task is common in the literature, but RSA is a dynamic measure, and moving window formats could provide more continuous, dynamic estimations of coregulation in line with prior work that has documented RSA concordance occurring in shorter time frames (e.g., Feldman et al. 2011). Another limitation is that, in some analytic models, the play and stress conditions were aggregated, which constrained our ability to analyze the cumulative effects of RSA concordance or lack thereof across the study period. Lastly, the cross‐sectional design of this study does not allow us to establish causal conclusions.

### 4.2 Implications and Conclusion

Findings from this study advance our understanding on the developmental correlates of mother–infant RSA coregulation, particularly in the context of adversity. These insights have important implications for future research and for strengthening intervention programming. First, much of the extant research on RSA coregulation has focused on samples without documented histories of adversity, often finding that mother–child RSA coordination is associated with positive child developmental outcomes. However, our findings suggest that families with experienced adversity may show different patterns of physiological coregulation that may signify resilience or adaptation in certain caregiving contexts. The growth models used in this study provide insight into how dynamic trajectories of physiological regulation unfold both at the individual level and within the dyad. Notably, infants’ RSA changes may contribute to variability in coregulation, particularly when developmental challenges limit their ability to maintain engagement as task demands shift in different caregiving contexts. This physiological variability may be further influenced by the relatively older infants and broad age range in this study, and could be driven by a subset of infants. For example, some RSA patterns in the present study differed from those reported in prior research using the double FFSF with younger infants (e.g., Ritz et al. 2012). Future analyses that incorporate the infant developmental stage could help clarify these effects.

Although our study is limited in its smaller sample size and cross‐sectional analysis, our findings contribute to understanding real‐time mother–infant physiological processes in relation to infant development among adversity‐exposed dyads. These findings build on prior research on RSA coregulation among mothers and preschool‐aged children (Lunkenheimer, Busuito, et al. 2018; Lunkenheimer, Tiberio, et al. 2018), highlighting specific patterns of physiological coregulation that first emerge during infancy and may persist into early childhood. Future research should consider longitudinal study designs with larger samples as well as including additional measures of the sympathetic nervous system to assess how adversity‐exposed mothers and infants coordinate their arousal in response to variations in maternal support and engagement. A key strength of this study is that data were collected in participants’ homes, which may have enhanced ecological validity by capturing mother–infant interactions in a familiar environment. Conducting the FFSF in the home may have alleviated added stress that is typically associated with laboratory settings, potentially allowing for a more accurate reflection of dyadic regulation processes as they occur in day‐to‐day interactions. Future studies could compare home‐ and lab‐based assessments to determine the extent to which the setting influences patterns of mother–infant physiological coregulation. Moreover, simultaneously including measures of affective and behavioral coregulation is an important direction for future research to better understand how multiple coregulatory processes operate together, which could support behavioral interpretations of physiological coregulation and refine understanding of relations between coregulation and children's developmental outcomes.

Our findings imply important future directions for intervention programs aimed at enhancing parenting support and infant development, particularly for higher‐risk families. Strengthening these programs could involve encouraging mothers to engage developmentally with their infants, regardless of the infants’ skill levels, as this engagement may foster better adaptation in challenging contexts. Furthermore, providing mothers with resources to help them self‐regulate during conditions that require varying levels of their support and engagement could be invaluable. By equipping mothers with strategies for self‐regulation, they may be better positioned to effectively support their children, especially those who have lower developmental milestones and social competence. This approach could lead to more positive outcomes for both mothers and infants, promoting healthier mother–infant interactions, even under adverse circumstances.

## Conflicts of Interest

The authors declare no conflicts of interest.

## Data Availability

This study was not preregistered. Analysis code may be available on request from the corresponding author. The data herein are not publicly available.
